# Acute T-cell lymphoblastic leukemia: chimeric antigen receptor technology may offer a new hope

**DOI:** 10.3389/fimmu.2024.1410519

**Published:** 2024-08-13

**Authors:** Jiajie Jing, Yuan Ma, Ziwen Xie, Bingyan Wang, Yueming Chen, Enjie Chi, Jiadong Wang, Kejin Zhang, Zhujun Wang, Sisi Li

**Affiliations:** ^1^ Department of Clinical Medicine, Hangzhou City University School of Medicine, Hangzhou, China; ^2^ Department of Pediatrics, Union Hospital, Tongji Medical College, Huazhong University of Science and Technology, Wuhan, Hubei, China

**Keywords:** T-ALL, pathogenesis, chimeric antigen receptor, CAR-T, CAR-NK

## Abstract

Acute lymphoblastic leukemia (ALL) is a prevalent malignancy affecting the hematopoietic system, encompassing both B-cell ALL (B-ALL) and T-cell ALL (T-ALL). T-ALL, characterized by the proliferation of T-cell progenitors in the bone marrow, presents significant treatment challenges, with patients often experiencing high relapse rates and poor long-term survival despite advances in chemotherapy and hematopoietic stem cell transplantation (HSCT). This review explores the pathogenesis and traditional treatment strategies of T-ALL, emphasizing the promising potential of chimeric antigen receptor (CAR) technology in overcoming current therapeutic limitations. CAR therapy, leveraging genetically modified immune cells to target leukemia-specific antigens, offers a novel and precise approach to T-ALL treatment. The review critically analyzes recent developments in CAR-T and CAR-NK cell therapies, their common targets, optimization strategies, clinical outcomes, and the associated challenges, providing a comprehensive overview of their clinical prospects in T-ALL treatment.

## 1 Introduction

T-cell acute lymphoblastic leukemia (T-ALL) predominantly originates from early T-cell progenitors, constituting 25% and 15% of adult and pediatric acute leukemias, respectively. The pathogenesis of T-ALL is intricate, and therapeutic options for T-ALL are comparatively restricted when compared to B-cell acute lymphoblastic leukemia (B-ALL) (1). Presently, newly diagnosed T-ALL patients commonly undergo high-risk multi-agent chemotherapy regimens lasting 2-3 years, with or without concurrent cranial radiotherapy. Despite novel drug combinations with chemotherapy have moderately improved the prognosis of T-ALL, achieving an 80% complete remission (CR) rate, over 50% of patients experience disease relapse and develop drug resistance. Even with allogeneic hematopoietic stem cell transplantation (allo-HSCT), the 5-year disease-free survival rate (5-DFS) for adult T-ALL remains below 30%, with approximately 30% cumulative relapse rate observed in pediatric T-ALL. The long-term survival rate for relapsed patients is less than 15% (2). HSCT stands as the sole potential curative option available at present (3). A pivotal prognostic factor for T-ALL is the presence of Minimal Residual Disease (MRD) after the induction and consolidation therapy phases. MRD denotes the persistence of a small number of leukemia cells in the patient’s body post-treatment, potentially predisposing to relapse (4). Common MRD markers used include flow cytometry markers (e.g., CD3, CD7, CD4, CD8, CD1a) and molecular markers (e.g., TCR gene rearrangements, NOTCH1 mutations) (5, 6).

The Chimeric Antigen Receptor (CAR) represents a revolutionary immunotherapy approach that uses genetic engineering to enable immune cells to specifically identify and eradicate tumor cells. This methodology revolves introducing receptor genes into immune cells via gene editing techniques, enable them to recognize tumor-associated antigens (TAAs). These modified cells are then expanded *in vitro* and administered to patients, facilitating the precise targeting and efficient elimination of tumor cells by the immune system. Currently, CAR immunotherapy predominantly harnesses T cells and NK cells as effector cells, with some partial involvement of B cells activated through cytokine networks. Particularly in hematologic malignancies, CAR-T immunotherapy, which employs T cells as effector cells, has demonstrated remarkable efficacy (7).

## 2 Pathogenesis and clinical treatment of T-ALL

### 2.1 Pathogenesis of T-ALL

**Figure 1:**
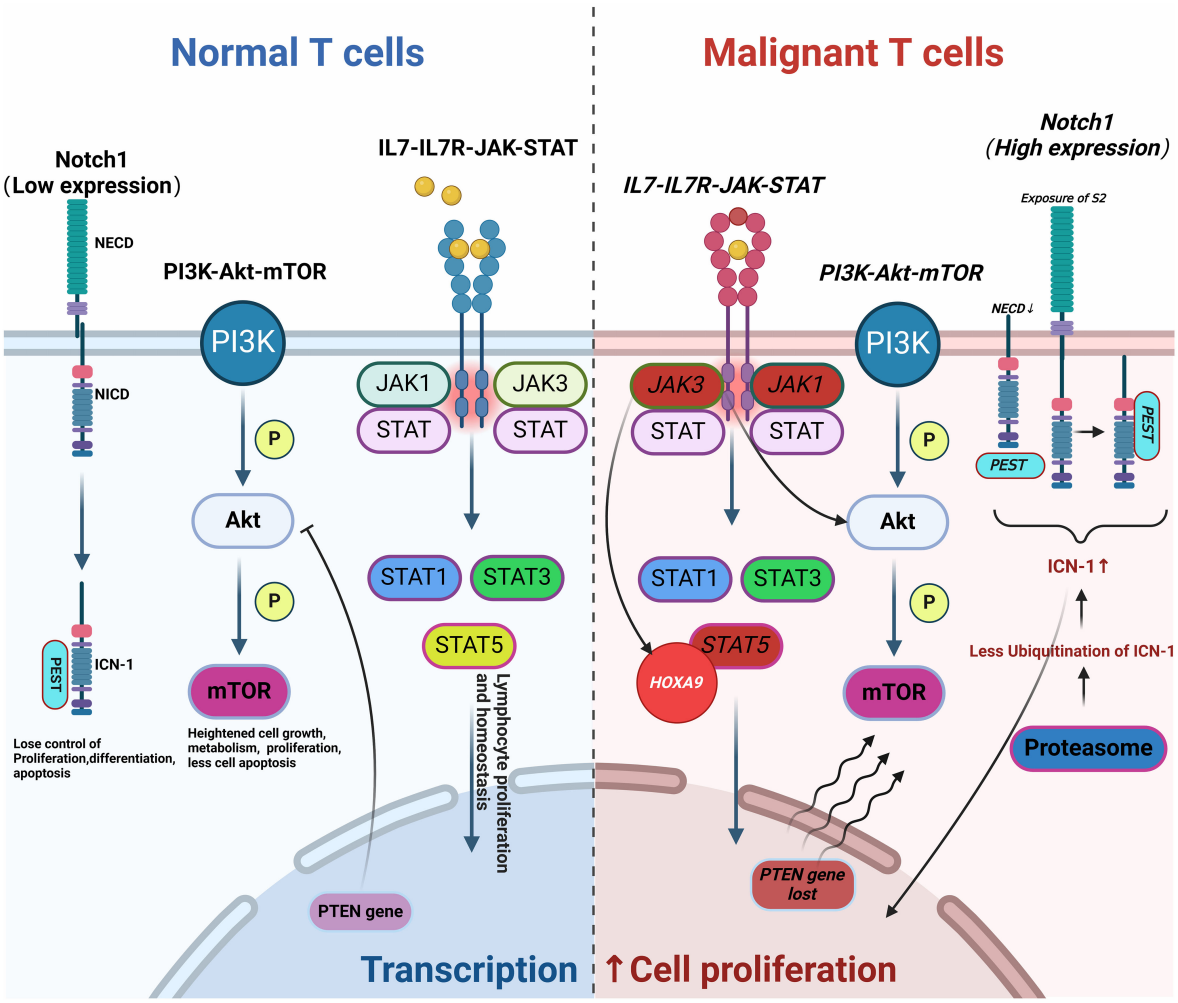
Pathogenesis of T-ALL. Abnormal changes in molecular pathways (such as NOTCH, IL7-IL7R-JAK-STAT, and PI3K-Akt-mTOR) lead to aberrant T cell proliferation and differentiation, resulting in disease onset, progression, and drug resistance (Malignant mutations are indicated in italics). This figure was drawn using Biorender.

T-ALL arises from the abnormal proliferation of T-lineage precursor cells within the bone marrow, influenced significantly by aberrant signaling pathways. The NOTCH, IL7-IL7R-JAK-STAT, and PI3K-Akt-mTOR pathways are critically implicated in T-ALL. Dysregulation in these pathways disrupts normal cellular processes, including metabolism, proliferation, differentiation, and apoptosis, ultimately leading to the development of T-ALL. These disruptions also play a significant role in contributing to drug resistance. For instance, NOTCH1 mutations, found in about 60% of T-ALL cases, lead to uninhibited signaling that promotes leukemic growth. These mutations can involve the disruption of the NOTCH1 gene itself or inactivating mutations of its negative regulators like FBXW7 (8–10). Additionally, NOTCH1 negatively regulates the tumor suppressor gene P53 in T-ALL through various mechanisms, such as inhibiting P53 activation, phosphorylation, and nuclear localization via eIF4E, leading to drug resistance in malignant cells. Mutations in the IL-7R and JAK-STAT pathway components are identified in 20-30% of T-ALL cases, promoting oncogenic activations that exacerbate the disease progression and mediate resistance to glucocorticoids (11, 12). The PI3K-Akt-mTOR pathway, often activated by the loss of tumor suppressor PTEN, is another critical factor of T-ALL pathogenesis, promoting malignant cell survival and resistance to therapy (13, 14). Epigenetic changes, such as DNA methylation and histone modifications, and dysregulation of apoptosis-related genes (e.g., TP53, BCL-2) further reduce sensitivity to chemotherapy. Genetic predispositions and environmental factors, such as exposure to ionizing radiation or carcinogenic chemicals, also contribute to the risk of developing T-ALL (15). Due to these complex pathogenic mechanisms, T-ALL is notably less responsive to chemotherapy than B-ALL. Targeted therapies developed from these insights, including inhibitors of γ-secretase, cyclin-dependent kinases, and mTOR, have shown promise in preclinical and clinical studies (3) (**Figure 1**).

### 2.2 Traditional clinical treatment

The management of T-ALL primarily revolves around combination chemotherapy, which is essential for induction, consolidation, maintenance therapy, and Central Nervous System (CNS) prophylaxis. The standard regimen includes drugs such as L-Asparaginase, Cyclophosphamide, Glucoroticoid and Vincristine. Intensive chemotherapy regimens are often employed to achieve effective induction and reduce relapse rates. To address CNS involvement, high-dose methotrexate and triple intrathecal therapy have proven effective in improving remission rates and managing CNS prophylaxis (16, 17). For high-risk patients, allo-HSCT is considered the most effective curative approach, particularly following initial intensive chemotherapy. Clinical trials have demonstrated that allo-HSCT provides superior outcomes compared to conventional chemotherapy or autologous transplantation, especially in standard-risk patients during the first CR (18, 19).

## 3 Studies on CAR technology in the treatment of T-ALL

CAR technology, which involves the modification of immune cells, has represented a significant breakthrough in the treatment of hematological malignancies. Notably, it has demonstrated remarkable success in treating drug-resistant B-ALL and B-cell non-Hodgkin lymphoma (NHL) (20).This technology has received approval from the US Food and Drug Administration (FDA) for clinical application (21–23). A CAR construct comprises three main domains: an extracellular domain, a transmembrane domain, and an intracellular activation fragment. The extracellular domain’s antigen-binding segment is responsible for precisely recognizing specific antigens on tumor cell surfaces, thereby facilitating targeted therapy. Meanwhile, the intracellular activation fragment activates effector cell killing functions upon antigen recognition. Different intracellular activation domains exhibit varying activation effects. The most commonly used activation domain is CD3ζ, which contains immunoreceptor tyrosine-based activation motifs (ITAMs) that initiate T-cell activation and proliferation upon antigen binding (24). CD3ζ alone can trigger cytotoxic responses but is often paired with co-stimulatory domains to enhance efficacy and persistence. FcRγ, an activation domain derived from the Fc receptor, is used in some CAR-NK cell therapies to initiate signaling cascades similar to CD3ζ in T-cells, thereby activating NK cells and inducing cytotoxicity against target cells (24, 25). Co-stimulatory domains such as CD28, 4-1BB (CD137), OX40 (CD134), and 2B4 (CD244) play crucial roles in enhancing CAR-T and CAR-NK cell therapies. CD28 provides essential secondary signals that enhance T-cell activation, proliferation, and cytokine production, but it may be associated with higher risks of CRS. 4-1BB enhances the survival and persistence of CAR-T cells by promoting anti-apoptotic signaling pathways, often showing longer persistence *in vivo* and lower incidences of severe cytokine release syndrome (CRS) compared to CD28-based CARs (26). OX40 enhances T-cell expansion and survival by activating the NF-κB pathway, increasing T-cell proliferation and resistance to apoptosis, thus improving the efficacy and durability of CAR-T cell responses (27), predominantly used in CAR-NK cell therapies, promotes NK cell activation and enhances cytotoxicity against target cells, sustaining NK cell responses and improving overall therapeutic efficacy (28). The choice of intracellular activation and co-stimulatory domains significantly impacts the efficacy, persistence, and safety profile of CAR-T and CAR-NK cell therapies. Understanding these differences allows for the optimization of CAR designs tailored to specific clinical needs. The following discussion will highlight therapeutic strategies that employ CAR technology in the treatment of T-ALL.

### 3.1 CAR-T therapy for T-ALL

**Figure 2:**
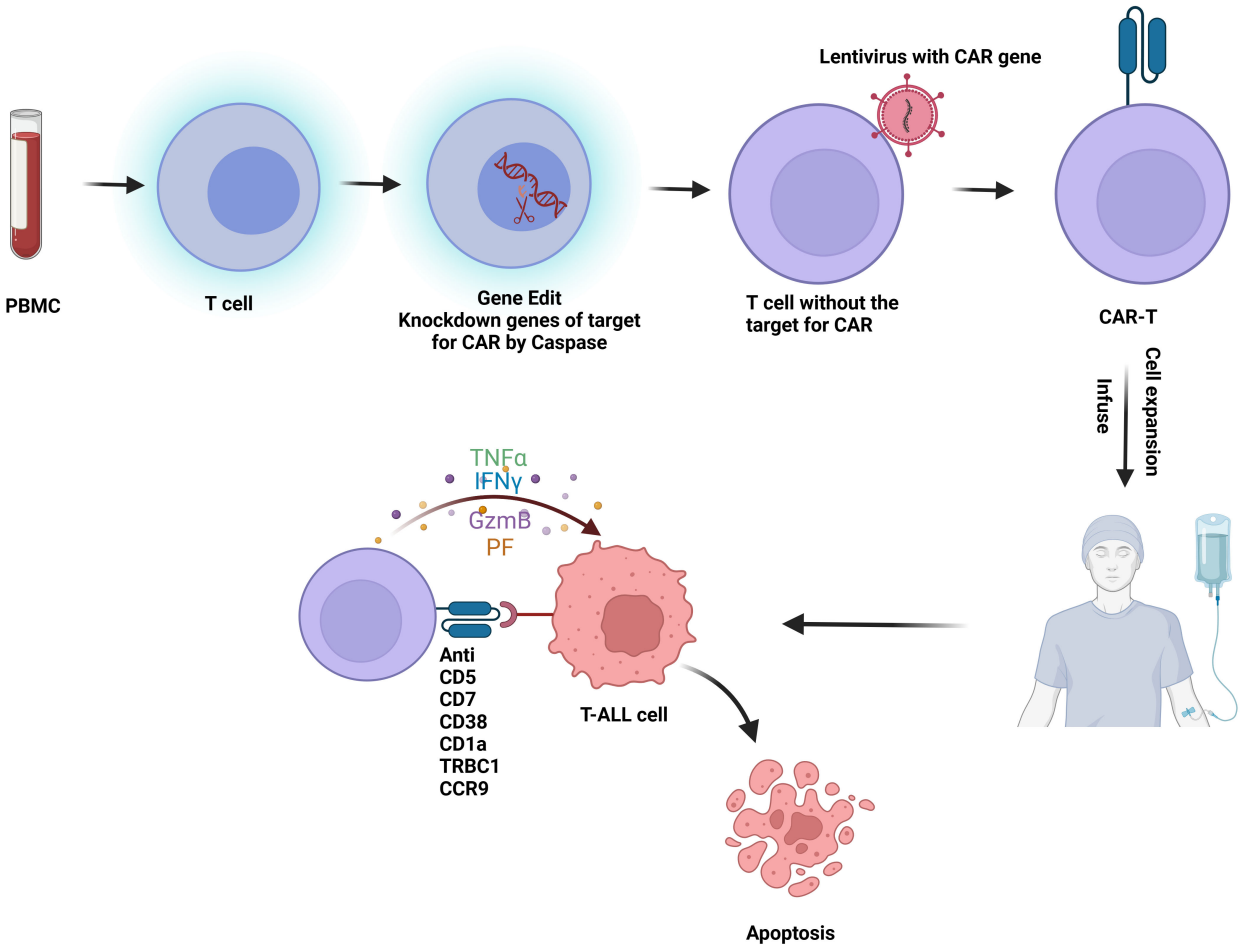
Flowchart of CAR-T therapy for T-ALL: T cells are first isolated from peripheral blood mononuclear cells (PBMC). These T cells undergo gene editing to silence the CAR-targeting antigen gene. Lentiviral vectors are then used to introduce the CAR construct into the T cells, creating CAR-T cells. Once engineered, the CAR-T cells are expanded and infused back into the patient, where they target and kill malignant T cells *in vivo*. This figure was drawn using Biorender.

CAR-T cell therapy involves genetically engineering a patient’s own T cells to target and destroy tumor cells (**Figure 2**). The primary challenge in developing CAR-T therapy for T-ALL is the presence of target antigens on both malignant and non-malignant T cells and effector cells. This dual expression can result in several complications (1): Damage to Normal T Cells: CAR-T cells may inadvertently attack normal T cells, leading to the depletion of healthy T cells and potentially causing severe immunodeficiency, posing a life-threatening risk (29) (2). Fratricide:Fratricide refers to CAR-T cells attacking each other due to the expression of the same target antigen. For example, in therapies targeting CD38 for T-ALL, CAR-T cells may also express CD38, leading to mutual destruction (30) (3). Contamination by malignant T Cells: During the isolation of therapeutic T cells, there is a risk of malignant T cells being mixed in and subsequently engineered into malignant CAR-T cells. Given these challenges, the progress of CAR-T cell therapy development for T-ALL has been hindered (31).

#### 3.1.1 CD5

CD5 is a characteristic surface marker found on malignant T cells, present in approximately 80% of cases of T-ALL and T-cell lymphomas (32). The Maksim’s group engineered a CD5-targeting CAR-T construct that includes an extracellular anti-CD5 single-chain variable fragment (scFv) derived from clone H65, an IgG Fc transmembrane domain, and intracellular segments from CD28 and TCRζ chains. Notably, CAR expression led to the down-regulation of CD5 on CAR-T cells, effectively reducing fratricide and enhancing *in vivo* expansion. Co-culture assays with CD5-positive T-ALL cell lines such as Jurkat, CCRF, and MOLT4 showed that CD5 CAR-T cells eliminated over 95% of target cells within 48 hours, with no detectable target cells after 7 days, indicating a high specificity and cytotoxic capability towards malignant T cells (33).

Further development by Dai’s group introduced fully human CD5-targeting heavy-chain variable (FHVH) domains to create a biepitopic CAR, termed FHVH3/VH1, which includes FHVH1 and FHVH3 domains that bind to different epitopes of the CD5 antigen. Additionally, they employed CRISPR-Cas9 technology to produce CD5 knockout (CD5^KO^) CAR-T cells, which reduces fratricide among the CD5 CAR-T population. This strategy not only enhanced the CD5 binding affinity of CAR-T cells but also significantly improved their therapeutic efficacy against CD5-expressing T-cell malignancies (34).

Moreover, integrating insights from the most recent advances, such as the development of a CH2CH3 hinge region that enhances the cytotoxicity of CD5 CAR-T cells (35) to optimize the efficacy and durability of CD5 CAR-T therapies. These enhancements not only bolster the CAR constructs’ direct anticancer activities but also refine their persistence in the hostile tumor microenvironment.

Overall, the evolving landscape of CD5 CAR-T cell research points to a promising future for the treatment of T-cell malignancies, with ongoing innovations aimed at improving efficacy, safety, and treatment accessibility (36).

#### 3.1.2 CD7

CD7 is highly expressed on the surface of T-ALL cells, making it a potent target for T-ALL therapy. Although CD7 is also found on NK cells and normal T cells, research in CD7 knockout mice has shown normal immune development, indicating that targeting CD7 does not impairing the essential functions of T and NK cells (37–41).

Diogo’s group used CRISPR-Cas9 to knock out CD7 expression in CAR-T cells, developed second-generation CAR-T cells incorporating CD28 and CD3ζ as intracellular signaling domains. These CD7 CAR-T cells demonstrated high efficacy in eliminating various T-ALL cell lines (Jurkat, CCRF-CEM, MOLT-4, Hut 78, SupT1, and Raji), as well as primary T-ALL cells, and exhibited therapeutic effects in mouse models of T-ALL (42). Jiang’s group employed a novel technique using EF1α to specifically disrupt the CD7 site on CAR-T cells. This approach aims to prevent fratricide, enhancing both the specificity and safety of CAR-T cell therapies (43). Conversely, Peihua Lu’s group adopted a different approach by employing a natural selection method to mask or sequence CD7 molecules on the cell surface using a specialized scFv, developing a novel CD7-targeting CAR (NS7CAR) -T cell. This method, though less invasive than gene editing, might not completely eliminate the risk of fratricide as the masking could be incomplete under certain physiological conditions. In a phase I trial (NCT04572308), NS7CAR was administered to 20 patients with refractory/relapsed T-ALL (r/r T-ALL) and T-cell lymphoblastic lymphoma (T-LBL). Nineteen patients achieved MRD negativity at Day28, attained complete remission, and became eligible for allo-HSCT (44). Additionally, Zhang’s group engineered CD7-targeted CAR-T cells that blocked CD7 expression by coupling CD7 nanoantibody VHH6 to an ER/Golgi-retention motif peptide. In a phase I clinical trial (NCT04004637), eight patients with r/r T-ALL received this treatment, achieving a CR rate of 87.5% at 3 months post-infusion, with detectable CAR-T cells persisting for up to 270 days (45).

Notably, Wong’s group introduced the (protein expression blocker) PEBL technology, which provides a groundbreaking solution to the problem of fratricide in CD7-targeted CAR-T therapy. By engineering a PEBL that retains CD7 within the Golgi apparatus, PEBL effectively prevents CD7 from being presented on the cell surface, thereby safeguarding CAR-T cells from fratricide. This approach not only maintains the efficacy of the CAR-T cells but also improves their survival and persistence in the patient’s body. The PEBL technology offers significant advantages over previous methods, including its non-invasive nature and the avoidance of potential gene editing complications. It represents a safer alternative that could potentially be applied to other CAR-T therapies targeting antigens expressed on the T cells themselves. A phase I is ongoing with these CAR T-cells in children and adults with relapse/refractory T-ALL in Singapore (CARTALL, NCT05043571). However, the scalability of this technology and its adaptability to other antigens require further investigation (46).

#### 3.1.3 C-C chemokine receptor type 9

CCR9, a G protein-coupled receptor with a 7-transmembrane structure, is expressed on approximately 5% of normal circulating T and B cells (47). Recent studies have demonstrated a significant upregulation of CCR9 in r/r T-ALL cases, compared to its minimal expression on normal T cells. This differential expression profile marks CCR9 as a viable target for CAR-T cell therapy for T-ALL. Paul’s team knocked out CCR9 of T cells and developed CCR9-targeted CAR-T cells. These CAR-T cells demonstrated strong cytotoxicity against both T-ALL cell lines and primary T-ALL blasts *in vitro*, confirming their potent anti-leukemic properties. Further exploration into the functionality of these CCR9-targeted CAR-T cells revealed significant therapeutic efficacy in preclinical studies, including standardized mouse models with T-ALL cell lines and patient-derived xenograft (PDX) models representing high tumor burden in r/r T-ALL (48). These studies confirmed that targeting CCR9 can lead to substantial reduction of tumor burden without causing significant toxicity to the normal T cell population.

#### 3.1.4 CD38

CD38, a cell surface glycoprotein part of the ribocylase family, is crucial in various cellular functions including cell migration, receptor-mediated adhesion, extracellular metabolism, and intracellular Ca2+ signaling. Its expression is significantly elevated in multiple myeloma (MM), NHL,T-ALL, and NK/T cell tumors, yet remains low in normal lymphocytes, bone marrow cells, and some non-hematopoietic tissues (49, 50). Importantly, CD38 is not present in multipotent hematopoietic stem cells, which are essential for bone marrow recovery, making it an attractive target for the treatment of various hematologic malignancies (51).

Wang’s group engineered T cells from PBMCs to express an anti-CD38 scFv fragment, controlled by an EF1α promoter and enhanced with co-stimulatory domains from 4-1BB and CD3ζ, developing a CD38 CAR-T. It demonstrated significant targeted killing capabilities against MM, B cell lymphoma, T-ALL, and NK/T cell lymphoma both *in vitro* and *in vivo* (52). Glisovic-Aplenc’s group further confirmed these findings, demonstrating the effectiveness of CD38 CAR-T in treating primary hematological malignant cells (53).

In 2023, Liao’s group introduced an innovative approach by integrating CD38-specific CARs into the CD38 gene locus itself, driven by different promoters. This novel approach led to the creation of both CAR-T and CAR-NK cells that exhibited robust anti-tumor activities. The use of native CD38 promoters for expression control potentially enhances the physiological relevance and tumor specificity of these CAR-modified cells, minimizing off-target effects and improving safety profiles (54).

#### 3.1.5 CD1a

CD1a is predominantly expressed on cortical T-ALL (coT-ALL) cells, but is absent on CD34-positive progenitor cells and mature T cells. This distinction provides a unique therapeutic advantage by minimizing off-target cytotoxic effects, significantly reducing the risk of damaging non-malignant hematopoietic cells (55–57).

Sanchez-Martinez’s group developed CD1a-targeting CAR-T cells, demonstrating strong and specific cytotoxicity when co-cultured with coT-ALL cell lines and primary lymphoid cells derived from coT-ALL patients. In NSG mouse xenograft models generated using Jurkat cells or T-ALL patient-derived tumor cells, it demonstrated significant anti-leukemic efficacy, with a marked reduction in tumor burden and prolonged survival in treated mice. These promising outcomes underscore the therapeutic potential of CD1a CAR-T cells and advocate for their further clinical development for this challenging leukemia subtype (58).

#### 3.1.6 TRBC1

Expression of T-cell receptor beta chain constant domain1 (TRBC1) and T cell receptor beta chain constant domain 2 (TRBC2) are mutually exclusive, with TRBC1 T cells comprising 25% to 47% of the T-cell population in healthy individuals. Maciocia’s group has made significant advancements in immunotherapy by developing a CAR-T therapy targeting the TRBC1. They engineered CAR-T cells that selectively recognize and eliminate TRBC1-expressing cells, both normal and malignant, while preserving TRBC2 positive cells, maintaining essential immune functions. This selective targeting strategy is advantageous over nonselective methods that deplete the entire T-cell population, thereby avoiding severe immunosuppression (59). However, a challenge identified by Zhang’s group highlights the complexity of CAR-T cell production: the anti-TRBC1 CAR gene might unintentionally transfer to malignant T cells during manufacturing, leading to a self-binding scenario where the CAR interacts with TRBC1 on malignant cells, thus shielding them from cytotoxic action (60). This underscores the need for meticulous control measures to prevent cross-contamination and ensure the purity of therapeutic cells.

In the evolving landscape of CAR-T cell therapy, it is essential to fully explore these technologies in larger, more diverse patient populations. Addressing challenges related to manufacturing, regulatory approval, and cost-effectiveness is equally important. Investigating PEBL and similar technologies in clinical trials will provide valuable insights into their long-term safety and efficacy, potentially revolutionizing treatment for T-ALL and other T-cell malignancies. Continuous innovations, like the integration of CAR constructs into specific gene loci demonstrated by Liao’s team, offer more regulated expression and could reduce the risk of cytokine release syndrome and other side effects. This advancement points to a future where CAR therapies are finely tuned for maximum efficacy and minimal toxicity. Targeting strategies focused on CD3, CCR7, CD4, and CD8 molecules have shown initial efficacy, broadening the scope of therapeutic targets. However, despite these advancements, challenges such as non-targeted cell damage and fratricide highlight the need for ongoing optimization. These developments underscore the dynamic and promising future of CAR cell therapy, aiming for greater precision and effectiveness in treating T-ALL and other T-cell malignancies (61).

### 3.2 CAR-NK therapy for T-ALL

**Figure 3:**
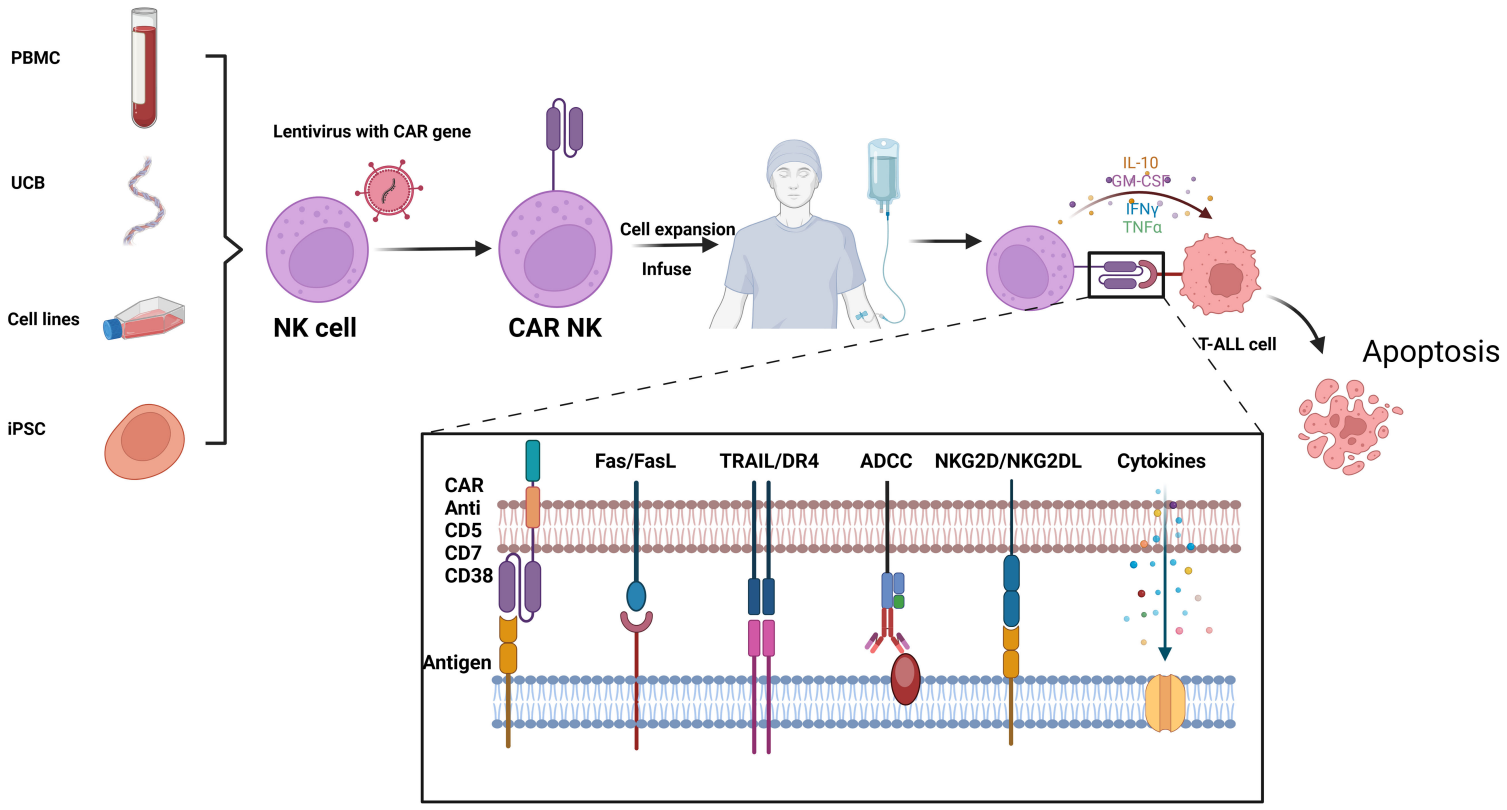
Flowchat and mechanistic diagram of CAR-NK therapy for T-ALL: NK cells from various sources are first isolated. Using lentiviral vectors, these NK cells are infected to create CAR-NK cells. Once engineered, the CAR-NK cells are expanded and infused back into the patient. *In vivo*, they target and kill malignant T cells through multiple mechanisms, including CAR-mediated targeting, Fas/FasL interactions, TRAIL/DR4 pathways, antibody-dependent cellular cytotoxicity (ADCC), and NKG2D/NKG2DL interactions. This figure was drawn using Biorender.

NK cells, also known as innate lymphoid cells, play a crucial role in the immune system by directly eliminating tumor cells without requiring specific activation. Unlike cytotoxic T cells, NK cells are not constrained by major histocompatibility complex (MHC) recognition and do not trigger the release of inflammatory cytokines such as IL-1 and IL-6 during killing, thereby avoiding the induction of CRS. In comparison to CAR-T cells, CAR-engineered NK cells offer several advantages, including potent anti-tumor activity, reduced toxicity and side effects, and a broader availability of cell sources (62). Similar to CAR-T therapy, the targeted molecules for CAR-NK cells include CD38, CD7, and CD5, etc. (**Figure 3**).

#### 3.2.1 CD38

As previously mentioned, Liao’s group integrated CD38-specific CARs into the CD38 gene locus, creating CAR-NK cells with native CD38 promoters that demonstrated robust anti-tumor activities and enhanced specificity while minimizing off-target effects. To address CD38 expression on normal NK cells and prevent fratricide, they employed a “two-in-one” CRISPR/Cas9 strategy, giving CAR-NK cells anti-fratricide capabilities. This allowed efficient elimination of primary T-ALL cells, showing the significant therapeutic effects (54). Furthermore, Clara’s group utilized CRISPR/Cas9 technology to disrupt CD38 expression in ex vivo expanded NK cells and integrated high-affinity CD16 into the edited CD38 locus. The integration of CD16 is based on its role as a high-affinity Fc receptor, which allows NK cells to bind to the Fc region of antibodies attached to target cells. This binding facilitates antibody-dependent cellular cytotoxicity (ADCC), enhancing the NK cells’ ability to identify and destroy malignant cells. This innovative approach not only circumvented NK cell autologous attack but also significantly enhanced antitumor efficacy by improving targeting and elimination of leukemic cells (63).

Looking forward, the potential of CD38 CAR-NK cells, with their targeted integration and controlled expression facilitated by CRISPR/Cas9, offers a promising alternative to conventional CAR therapies. However, the real-world efficacy of these cells requires comprehensive clinical trials to assess their stability, persistence, and effectiveness across various tumor environments.

#### 3.2.2 CD5

CD5 is recognized as a critical characteristic marker of T-ALL cells and is expressed on nearly all normal T cells, yet is absent on NK cells. This distinct expression profile enables the utilization of CD5 CAR-NK cell immunotherapy to bypass the fratricide effects often seen with CAR-T therapies. Xu’s group harnessed NK-92 cells as a platform for CD5-CAR modification, engineering anti-CD5 CAR constructs that incorporated different costimulatory domains: one with the T-cell-associated 4-1BB (BB.z) activating receptor and another with the NK cell-associated 2B4 (2B4.z) activating receptor. *In vitro* studies showed that both BB.z-NK and 2B4.z-NK cells exhibited targeted cytotoxicity against CD5-positive malignant cell lines, primary malignant cells, and normal T cells. In a T-ALL mouse xenograft model, both types of CAR-NK cells demonstrated significant anti-tumor effects, with 2B4.z-NK cells displaying enhanced anti-CD5 activity compared to BB.z-NK cells (64).

Further analysis by Voynova’s group compared the efficacy of CD3/CD5-directed CAR-NK and CAR-T therapies in T-ALL. Their findings highlighted that NK cells, when used as effector cells, effectively reduced the fratricide effect and addressed complications related to T cell aplasia and long-term immunodeficiency resulting from targeting molecules expressed on healthy T cells. CD3/CD5-directed CAR-NK cells do not cause long-term immunodeficiency due to their distinct biological characteristics and shorter lifespan compared to T cells. Unlike T cells, NK cells have a reduced risk of prolonged immune suppression and do not undergo extensive clonal expansion or persistence, minimizing their impact on long-term immune function. In contrast, CAR-T cells, being part of the adaptive immune system, can persist and expand over time, potentially leading to sustained depletion of target cells and subsequent immunodeficiency. Voynova’s group observed that although CD3 CAR-NK cells demonstrated robust efficacy against lymphoma cells *in vitro*, their performance in xenograft tumor models was compromised due to significant down-regulation of target antigens on malignant cells after the infusion of CD3 CAR-NK cells (65). In contrast, therapies directed towards CD5 present a potentially safer and more effective alternative. The addition of DAP10 co-stimulation imparts memory-like features to CAR-NK cells targeting CD5, enhancing their efficacy and persistence. This design significantly bolsters the therapeutic potential of CD5 CAR-NK cells as a treatment for T-ALL (66, 67).

#### 3.2.3 CD7

Zhu’s group developed CD7 CAR-NK-92MI cells, which exhibited significant cytotoxicity against CD7-positive hematological malignancies, including T-ALL, AML, and T-cell lymphoma, compared to their non-modified counterparts. These modified NK cells exhibited increased secretion of cytokines (e.g., IL-2, IFN-γ, granzyme B) play a critical role in mediating immune responses and enhancing cytotoxic activities (68). Jiang’s group developed a CD7-targeting CAR-NK by integrating the CAR directly into the CD7 gene locus of NK cells, using EF1α as the intracellular activation segment. This approach prevents the elimination of NK cells caused by their own CD7 expression, effectively addressing issues related to cell fratricide. These CD7-specific CAR-NK cells exhibit promising anti-tumor activity with reduced risk of off-target effects and enhanced tumor specificity (43).

## 4 Clinical studies of CAR technology in T-ALL

The clinical efficacy of CAR-T therapy in treating hematological malignancies has been well-established, with many related clinical trials currently underway. The number of trials investigating CAR-T cell therapy for T-ALL is steadily increasing, reflecting the rapid advancements in this field. Although CAR-NK therapy is under investigation for AML and solid tumors, its potential for treating T-ALL remains underexplored. While there is a comprehensive list of registered clinical trials for CAR-T cell therapy targeting T-ALL on the ClinicalTrials.gov website (refer to **Table 1**), there are no registered trials specifically focusing on CAR-NK cells for T-ALL. This highlights the emerging interest in CAR-NK cell therapy, necessitating further research to understand its potential in treating T-ALL.

**Table 1:** Clinical study of chimeric antigen receptor technology in the treatment of T-ALL (Data sources： ClinicalTrials.gov).

ClinicalTrialNo.	Target	Originating agency	CAR-T/NK	Starting time	Type of vector	Co-stimulatory domain
NCT05596266	CD5	Xuanwu Hospital, Beijing	CAR-T	2022/10/25	Lentiviral	N/A
NCT04594135	CD5	iCell Gene Therapeutics	CAR-T	2020/12/1	Lentiviral	N/A
NCT05487495	CD5	Beijing Boren Hospital	CAR-T	2022/7/27	N/A	N/A
NCT05043571	CD7	National University Hospital, Singapore	CAR-T	2021/9/14	Lentiviral	4-1BB,CD3ζ
NCT06136364	CD7	Heibei Senlang Biotechnology Inc., Ltd.	CAR-T	2023/8/15	N/A	N/A
NCT04572308	CD7	Hebei Senlang Biotechnology Inc., Ltd.	CAR-T	2020/10/1	Lentiviral	4-1BB,CD3ζ
NCT04934774	CD7	iCell Gene Therapeutics	CAR-T	2020/12/1	Lentiviral	N/A
NCT05620680	CD7	Shenzhen University General Hospital	CAR-T	2022/10/1	N/A	N/A
NCT04860817	CD7	920th Hospital of Joint Logistics Support Force of People’s Liberation Army of China	CAR-T	2021/12/1	N/A	N/A
NCT05290155	CD7	Shanghai General Hospital, Shanghai Jiao Tong University School of Medicine	CAR-T	2022/5/4	N/A	N/A
NCT05909527	CD7	Guangzhou Bio-gene Technology Co., Ltd	CAR-T	2023/5/1	N/A	N/A
NCT04004637	CD7	PersonGen BioTherapeutics (Suzhou) Co., Ltd.	CAR-T	2019/8/25	N/A	N/A
NCT04984356	CD7	Wugen, Inc.	CAR-T	2021/7/30	N/A	N/A
NCT05212584	CD7	iCell Gene Therapeutics	CAR-T	2022/1/28	Lentiviral	N/A
NCT04785833	CD7	PersonGen BioTherapeutics (Suzhou) Co., Ltd.	CAR-T	2021/3/8	Lentiviral	CD28,4-1BB
NCT04938115	CD7	Hebei Senlang Biotechnology Inc., Ltd.	CAR-T	2021/6/24	Lentiviral	4-1BB,CD3ζ
NCT05626400	CD7	Hebei Senlang Biotechnology Inc., Ltd.	CAR-T	2022/11/23	Lentiviral	4-1BB,CD3ζ
NCT05716113	CD7	Zhejiang University	CAR-T	2023/2/8	N/A	N/A
NCT05902845	CD7	Anhui Provincial Hospital	CAR-T	2023/6/15	N/A	N/A
NCT05398614	CD7	Hebei Senlang Biotechnology Inc., Ltd.	CAR-T	2022/3/1	N/A	N/A
NCT05885464	CD7	Beam Therapeutics Inc.	CAR-T	2023/6/2	N/A	N/A
NCT06064903	CD7	Bambino Gesù Hospital and Research Institute	CAR-T	2023/10/3	N/A	N/A
NCT05454241	CD7	Institute of Hematology & Blood Diseases Hospital, China	CAR-T	2022/7/12	N/A	N/A
NCT05377827	CD7	Washington University School of Medicine	CAR-T	2022/5/17	N/A	N/A
NCT05509855	CD7	Wugen, Inc.	CAR-T	2022/8/22	N/A	N/A
NCT04033302	CD7	Shenzhen Geno-Immune Medical Institute	CAR-T	2019/7/26	Lentiviral	N/A
NCT05995028	CD7	Shenzhen Geno-Immune Medical Institute	CAR-T	2023/8/16	N/A	N/A
NCT05745181	CD1a	The Affiliated Hospital of Xuzhou Medical University	CAR-T	2023/2/1	N/A	N/A
NCT05679895	CD1a	OneChain Immunotherapeutics	CAR-T	2023/1/31	Lentiviral	N/A
NCT05277753	multi-antigen	Shenzhen Geno-Immune Medical Institute	CAR-T	2022/3/14	N/A	N/A

N/A, Not available.

Although CAR-T cell therapy has shown positive effects in clinical trials of T-ALL, serious side effects may exist. For example, CRS, immune effector cell-associated neurotoxicity syndrome (ICANS) and the accompanying organ damage have become important reasons that prevent the clinical application of CAR-T (69). Among them, CRS is often described as the most serious complication of CAR-T therapy and poses a great threat to life (70, 71). The clinical features of CRS are high fever, sinus tachycardia, hypotension, hypoxia, cardiac dysfunction and other organ dysfunction (22, 70, 72–77). It is generally believed that CRS is started by excessive secretion of proinflammatory cytokines, mainly IFN-γ and TNF-α, when CAR-T cells are cytotoxic to tumor cells. Inflammatory cytokines further activate the interaction of natural killer cells, endothelial cells, monocytes/macrophages and dendritic cells, and further increase the secretion of cytokines, including IL-6, IL-1, IL-5, IL-10, etc., through an ever-amplifying cascade, eventually resulting in a severe systemic inflammatory response (73). Available clinical trial data indicate that the first symptom of CRS is often fever (75, 78, 79), which usually occurs hours to days after CAR-T cell infusion. After first fever, patients often exhibit circulatory and respiratory disorders, such as sinus tachycardia, low blood pressure, and hypoxia. Subsequently, nephrotoxicity and hepatotoxicity are common manifestations of organ toxicity (70). Due to the rapid onset, for acute and severe CRS, medical staff may need to firstly give corresponding emergency strategies, such as vasopressors to relieve hypotension, rather than giving cytokine antagonists only (80). Hypoxia caused by capillary leak syndrome caused by CRS can be fatal in some cases. In critical cases, mechanical ventilation should be adopted decisively (81–83).

Compared with CRS, ICANS has a slightly better prognosis. In most cases, ICANS is completely reversible (84), but fatal cerebral edema and epilepsy still occur, suggesting that ICANS is still a serious complication of CAR-T therapy, which needs extensive attention (85, 86). The onset cycle of ICANS was variable, ranging from day 2 (77) to week 4 (87) after CAR-T infusion, suggesting that long-term, scheduled observation of neurotoxicity may be needed after CAR-T treatment. ICANS beyond the CNS, delirium, hallucinations, cognitive impairment, tremor, ataxia, speech disorders, nerve palsy, focal motor or sensory dysfunction, myoclonus, sleepiness, dull, or seizures (88, 89) were observed in clinical trials. There is now clear evidence that ICANS is closely related to systemic cytokine levels and CRS exacerbation. Among them, IFN-γ, IL-15, IL-6, IL-10, GM-CSF, IL-2, IL-2Rα, IL-1RA, and CXCL10 are considered to be associated with ICANS. However, the pathophysiological link remains unclear, as not all patients with severe CRS develop ICANS, and not all patients with ICANS experience CRS (84). Therefore, it is more accurate to describe ICANS as a process that is associated with the onset of CRS but operates independently of it (88). The present studies have pointed out that, in addition to clinical necessary symptomatic first aid measures, favorable effects have also been reported for blockers targeting cytokines. For example, IL-1 blockers can alleviate the occurrence of macrophage-mediated CRS and can reduce the severity of CAR-T cell-related neurotoxicity (90). Tocilizumab and/or single-dose corticosteroids have a good remission effect in CRS caused by CAR-T, but long-term use is prone to develop drug resistance (88). In addition to drug therapy to alleviate CRS, modifying the intracellular signaling domains of CAR-T cells can also achieve this goal. Studies have shown that the toxicity rate of CAR-T containing 4-1BB is higher than that of CD28-containing CAR-T (91).

Graft-versus-host disease (GVHD) is a severe immune response that occurs when donor CAR-T cells attack the recipient’s tissues, typically seen in allogeneic CAR-T cell therapies. This condition arises when T lymphocytes from the allogeneic donor graft react against the recipient’s antigens after transplantation, potentially leading to severe complications. To mitigate GVHD, strategies include using autologous CAR-T cells or modifying CAR constructs to reduce immune responses (92, 93).

Currently, the application of CAR-T cell therapy in T-ALL is in its early stages. Initial clinical trials and studies have shown promising early response rates, including complete remission in r/r T-ALL patients. However, long-term follow-up data remains limited, with most studies only covering periods of several months to a few years, making it premature to draw conclusions on long-term efficacy and safety. While some patients maintain prolonged disease-free survival after initial remission, relapse does occur, indicating the need for further research. Numerous clinical trials are ongoing globally, and as more patients are treated and followed up, more comprehensive long-term data will emerge. Thus, while early results are encouraging, more research and long-term data are necessary to make definitive conclusions about the long-term outcomes of CAR-T therapy in T-ALL (44, 94, 95).

## 5 Comparison of different CAR technologies in the treatment of T-ALL

CAR-T and CAR-NK therapies each have unique strengths and challenges, particularly in treating T-ALL. CAR-T therapies are celebrated for their potent antitumor efficacy and have demonstrated remarkable results in clinical settings. However, major clinical challenges such as CRS, neurotoxicity, GVHD and off-target effects limit their application and efficacy. The intricate individualized preparation process of CAR-T cells further complicates their widespread use (96–98).

CAR-NK cell therapy also presents unique advantages. CAR-NK cells can be derived from allogeneic sources and used as off-the-shelf therapeutics without the risk of inducing GVHD, offering a versatile and potentially safer treatment option. NK cells naturally possess an ability to eliminate tumor cells via diverse activating receptors, and when coupled with the specific tumor-killing effect mediated by CARs, this provides a dual mechanism for effective tumor therapy (99). Additionally, CAR-NK cells are associated with fewer and less severe instances of CRS due to their distinct activation pathways. The development of off-the-shelf CAR-NK cells also presents economic benefits, reducing the need for individual patient cell harvesting and customization (96–98). The diversity in the sources of NK cells—such as peripheral blood, umbilical cord blood, human embryonic stem cells (hESCs), induced pluripotent stem cells (iPSCs), and NK cell lines like NK-92—streamlines the preparation process and mitigates costs associated with therapy (100). However, the persistence and expansion of CAR-NK cells *in vivo* remain challenges that need to be addressed to enhance their therapeutic efficacy.

In conclusion, the ongoing comparative analysis and refinement of CAR-T and CAR-NK platforms are crucial for enhancing their therapeutic index. This involves advancing the design and functionality of CAR constructs and deepening our understanding of the tumor microenvironment to support the efficacy of these engineered cells. As the field progresses, integrating insights from clinical trials and translational research will be essential to optimize the use of CAR-based therapies in oncology, ensuring they deliver the most effective and safest treatments possible.

## 6 Conclusion and Outlook

This review has explored the pathogenesis and conventional treatment modalities of T-ALL, emphasizing the latest advancements in CAR technology for its management. While CAR-T cell therapy has demonstrated remarkable efficacy, its application is often limited by potential side effects. In contrast, CAR-NK cell therapy offers unique advantages, such as improved safety and the potential for off-the-shelf use. The continuous and rapid advancements in CAR-T and CAR-NK technology encompass both improvements in the technology itself and its combination with other approaches. Moreover, dual-antigen activated synNotch CARs have shown significant advantages in both solid tumors and hematological malignancies, offering new strategies for better tumor targeting and reducing off-target effects (101, 102).

In summary, the evolution of CAR technology instills renewed optimism for the precise treatment of T-ALL. While challenges persist, ongoing technological innovations and optimizations are expected to enhance the efficacy and safety of CAR-based immunotherapy for T-ALL patients. Future research should focus on refining CAR design, target selection, and managing associated side effects to maximize therapeutic benefits while minimizing adverse reactions. This concerted effort aims to establish CAR therapies as precise therapeutic tools with substantial promise for T-ALL management.
